# Ecosystem engineers can regulate resource allocation strategies in associated plant species

**DOI:** 10.3389/fpls.2024.1387951

**Published:** 2024-06-06

**Authors:** Pengfei Yang, Mengqiu Niu, Quansheng Fu, Lishen Qian, Meihong Huang, Zhimin Li, Hang Sun, Jianguo Chen

**Affiliations:** ^1^ School of Life Science, Yunnan Normal University, Kunming, Yunnan, China; ^2^ Key Laboratory for Plant Diversity and Biogeography of East Asia, Kunming Institute of Botany, Chinese Academy of Sciences, Kunming, Yunnan, China; ^3^ School of Ecology and Environment, Southwest Forestry University, Kunming, Yunnan, China; ^4^ College of Biodiversity Conservation, Southwest Forestry University, Kunming, Yunnan, China

**Keywords:** alpine ecosystem, facilitation, reproductive effort, resource allocation, root fraction, trade-off

## Abstract

Balancing the biomass requirements of different functions for the purpose of population reproduction and persistence can be challenging for alpine plants due to extreme environmental stresses from both above- and below-ground sources. The presence of ecosystem engineers in alpine ecosystems effectively alleviates microenvironmental stresses, hence promoting the survival and growth of other less stress-tolerant species. However, the influence of ecosystem engineers on plant resource allocation strategies remains highly unexplored. In this study, we compared resource allocation strategies, including biomass accumulation, reproductive effort (RE), root fraction (RF), as well as relationships between different functions, among four alpine plant species belonging to Gentianaceae across bare ground, tussock grass-, cushion-, and shrub-engineered microhabitats. Shrub-engineered microhabitats exerted the strongest effects on regulating plant resource allocation patterns, followed by tussock grass- and cushion-engineered microhabitats. Additionally, apart from microhabitats, population background and plant life history also significantly influenced resource allocation strategies. Generally, plants established within engineered microhabitats exhibited higher biomass accumulation, as well as increased flower, leaf and stem production. Furthermore, individuals within engineered microhabitats commonly displayed lower RF, indicating a greater allocation of resources to above-ground functions while reducing allocation to root development. RE of annual plants was significantly higher than that of perennial plants. However, individuals of annual plants within engineered microhabitats showed lower RE compared to their counterparts in bare ground habitats; whereas perennial species demonstrated similar RE between microhabitat types. Moreover, RE was generally independent of plant size in bare-ground habitats but exhibited size-dependency in certain populations for some species within specific engineered microhabitat types. However, size-dependency did exist for absolute reproductive and root biomass allocation in most of the cases examined here. No trade-offs were observed between flower mass and flower number, nor between leaf mass and leaf number. The capacity of ecosystem engineers to regulate resource allocation strategies in associated plants was confirmed. However, the resource allocation patterns resulted synergistically from the ecosystem engineering effects, population environmental backgrounds, and plant life history strategies. In general, such regulations can improve individual survival and reproductive potential, potentially promoting population persistence in challenging alpine environments.

## 1 Introduction

In alpine ecosystems, environmental conditions, encompassing air and soil temperature oscillation, soil moisture and nutrient supply, sunlight exposure, and wind speed, generally impose stress on plant establishment and/or survival (Körner, 2003; Nagy and Grabherr, 2009). However, various types of ecosystem engineers (Jones et al., 1994), such as shrubs, cushions, and tussock plants in alpine ecosystems, effectively mitigate the severity of these environmental challenges (referred to as ‘ecosystem engineering effects’) for other less stress-tolerant species (Perelman et al., 2003; Badano and Cavieres, 2006a; Cavieres et al., 2007, 2006b; Anthelme et al., 2014; Ballantyne and Pickering, 2015; Chen et al., 2015b, 2019, 2020b; Ramírez et al., 2015; Zhang et al., 2018). The microhabitats created by these ecosystem engineers, hereafter referred to as ‘engineered microhabitats’, are typically characterized by benign conditions with abundant resources that provide a safer and more suitable environment for other species. Consequently, such engineered microhabitats can exert significant influences on various aspects of plant communities (Badano and Cavieres, 2006a, 2006b; Cavieres et al., 2007; Brooker et al., 2008; Ballantyne and Pickering, 2015; Chen et al., 2015b; Vega-Alvarez et al., 2019).

The accumulation and allocation of resources into different functional organs in plants have significant implications for ecological and evolutionary processes (Weiner, 1988, 2004; Reekie and Bazzaz, 2005; Bonser and Aarssen, 2009; Salguero-Gómez et al., 2016). Plants can adjust their resource allocation strategies in response to environmental changes to ensure an optimal development of each function, as suggested by the optimal partitioning theory (OPT) (Bloom et al., 1985; Chapin et al., 1987). Various factors, including abiotic factors such as water, nutrient, and light availability, as well as biotic factors like sexual morphs, individual size, and competition, can affect plant resource absorption and allocation strategies (Méndez and Karlsson, 2004; Poorter et al., 2012; Chen et al., 2016, 2017; Oram et al., 2023; Wang et al., 2023). According to the OPT, plants allocate more resources gained from the surrounding environment to organs that experience stronger constraints (Bloom et al., 1985; Chapin et al., 1987). For example, under soil water or nutrient stress conditions, plants may allocate more resources toward developing robust root systems for accessing deep soil water/nutrients (Ma et al., 2021; Feng et al., 2023). On the contrary, under strong light stress caused by competition with neighboring plants or shading from surrounding objects, plants can allocate more resources toward above-ground components, such as taller stems and/or increased leaf production (Poorter et al., 2012; Chen et al., 2020a). Considering the inherent limitations of available resources, particularly in challenging environments, allocating additional resources to certain organs inevitably results in a reduction of resources available for other organs. Trade-offs between different functional organs are thus an integral part of plant resource allocation processes (Forbis and Doak, 2004; Du and Qi, 2010; Buckley and Avila-Sakar, 2013; Rosa-Schleich et al., 2019; Umaña et al., 2021). Given the constraints imposed by extreme environments on alpine plants (Körner, 2003; Nagy and Grabherr, 2009), certain species have evolved to allocate additional resources toward the development of specialized organs that can effectively withstand local harsh conditions (Sun et al., 2014 and references therein). For example, *Saussurea medusa* (Asteraceae) exhibits dense trichomes, while *Rheum nobile* (Polygonaceae) possesses large bracts, both of which serve to elevate temperatures within inflorescences/infructescences, thus safeguarding flowers against external damages and promoting seed development (Yang et al., 2008; Yang and Sun, 2009; Song et al., 2013). Additionally, many alpine plants develop robust root systems to ensure stability in unstable substrates or facilitate nutrient and water absorption from deep soils (Hodge, 2010). In accordance with trade-off theory, such additional costs may lead to reduced allocation towards reproductive functions (Reekie and Bazzaz, 2005).

However, as mentioned above, ecosystem engineers can create microhabitats that provide less stressful conditions and richer resources compared to bare-ground habitats, which act as refuges for other less stress-tolerant plants (Badano and Cavieres, 2006a, 2006b; Cavieres et al., 2007; Chen et al., 2015a, 2015b, 2019, 2020b; Ramírez et al., 2015). Theoretically, plants established within these engineered microhabitats may not need to allocate extra resources to resist severe environments like their counterparts in bare-ground habitats do. According to the OPT (Bloom et al., 1985; Chapin et al., 1987), it is reasonable to predict that the saved resources could be reallocated towards other functions, either vegetative or reproductive. This reallocation is particularly crucial for annual plants, which have a limited growing season and need to complete their life cycles quickly without investing heavily in resistance mechanisms. Consequently, if they are relieved from the necessity of allocating additional resources towards resistance against harsh environments, they can reallocate these saved resources towards reproductive functions instead. In contrast, perennial species, which have multiple years to complete their life cycles, may accumulate sufficient resources before reproduction becomes a priority.

**Figure 1:**
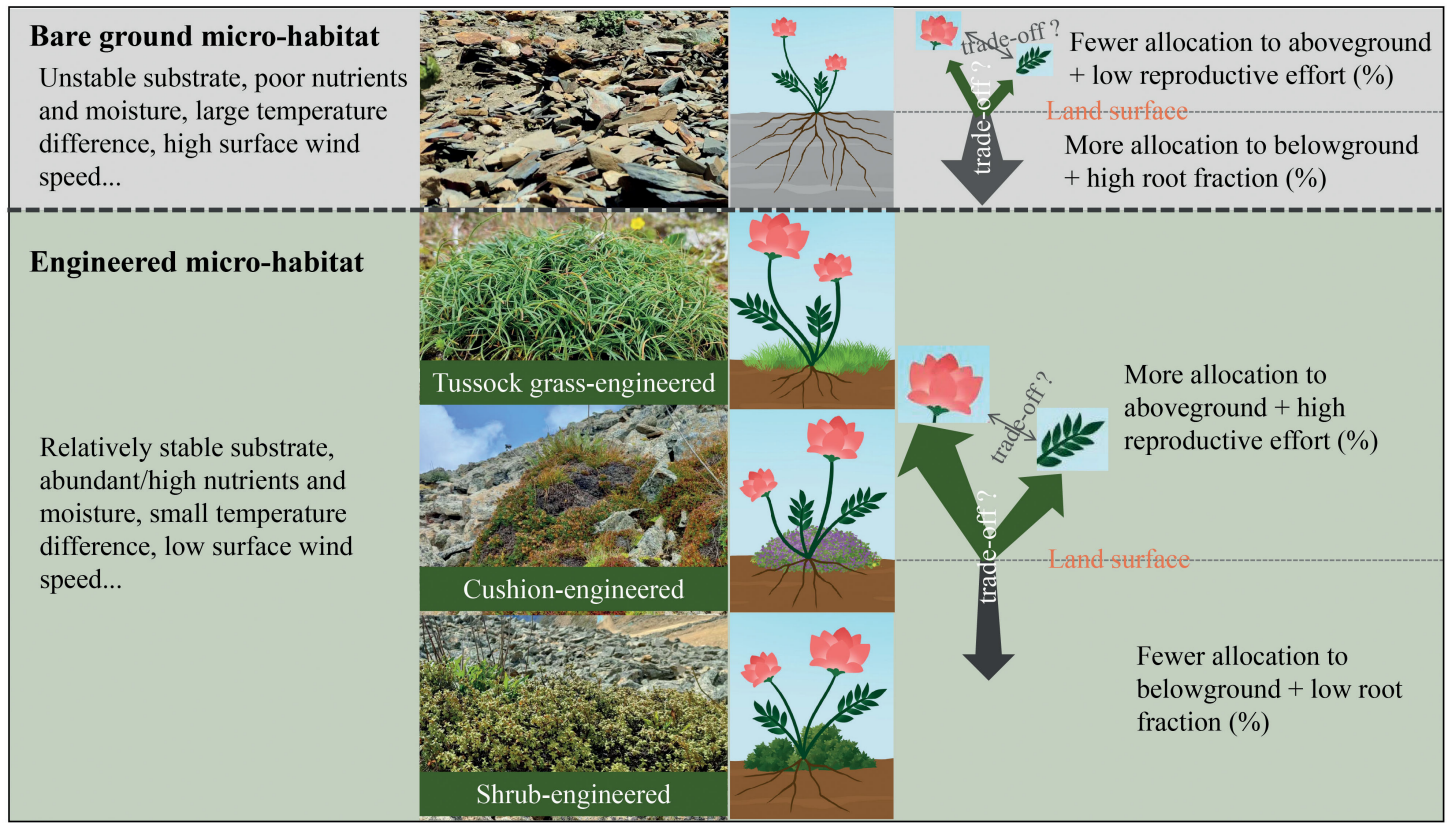
Scientific principle and hypotheses presented in this study. Compared to bare-ground habitats, engineered microhabitats mitigate specific environmental stresses, particularly those occurring below-ground, for associated plant species. Consequently, the allocation of additional resources by these plants to combat relevant stresses may not be necessary or reduced, resulting in a lower root fraction. The saved resources are thus hypothesized to be reallocated towards other functions, resulting in an enhanced reproductive effort.

In this study, we investigated how four alpine plant species belonging to Gentianaceae allocate resources across different microhabitats—bare-ground, shrub-, tussock grass-, and cushion-engineered microhabitats. These life forms, including shrubs, cushions, and tussock grasses, are recognized globally as ecosystem engineers that modify microenvironmental conditions, thus benefiting other plant species (Badano and Cavieres, 2006a, 2006b; Cavieres et al., 2007; Anthelme et al., 2014; Ballantyne and Pickering, 2015; Chen et al., 2015a, 2015b, 2019, 2020b). Our study area, similar to other alpine regions, features shrub and cushion plants that significantly alter the local microclimate (Yang et al., 2010; Chen et al., 2015a, 2015b, 2019, 2020b). Here, we considered ‘sedge aggregations’ (hereafter referred to as ‘sedge grass’ or ‘grass’), which are small patches primarily composed of sedge plants (**Figure 1**), as another form of engineered microhabitat. Sedge species, known for forming dense tussocks through vegetative reproduction, can ameliorate environmental conditions at a microscale level (Peach and Zedler, 2006).

Specifically, we address three main questions and relevant hypotheses as follows: *і*) Do ecosystem engineers enhance the growth of target plants? Given that engineered microhabitats provide a less severe environmental condition (**Figure 1**), we hypothesize that plant individuals established within these habitats will exhibit better performances compared to their counterparts in bare-ground habitats where they are exposed to more severe environments. *ii*) How do ecosystem engineers influence plant resource allocation strategies? Plant individuals established within engineered microhabitats may allocate fewer resources to stress resistance and instead invest more into reproductive functions. Therefore, we hypothesize that individuals established within engineered microhabitats will display higher reproductive efforts (*i.e.*, a greater fraction of total resources allocated to reproduction, as defined by Williams, 1966; **Figure 1**). Furthermore, since different ecosystem engineers can modify microenvironments in distinct ways (Chen et al., 2015a, 2019), resulting in varying types and magnitudes of stresses experienced by associated plants, we propose that the modulation of plant resource allocation strategies by ecosystem engineers differs among different types of engineered microhabitats. *iii*) Do trade-offs exist between different functions of target plants, and how does the presence of ecosystem engineers influence these trade-off patterns? Ecosystem engineers can enhance soil nutrient and moisture levels (Cavieres et al., 2007; Yang et al., 2010; Chen et al., 2015b, 2020b; **Figure 1**), potentially reducing stresses from below-ground conditions within engineered microhabitats. Consequently, we hypothesize that target plants will allocate more resources towards above-ground functions, especially in annual plants, resulting in a lower root fraction (proportion of resources allocated to roots) compared to bare ground habitats (**Figure 1**). Given the inherently poor development of alpine soils (Körner, 2003), which implies limited total resource availability in alpine ecosystems, trade-offs may indeed occur between various functions of the target plants. However, since engineered microhabitats can modify resource availability, they may also influence the trade-off patterns.

## 2 Materials and methods

### 2.1 Study species and populations

Four species of Gentianaceae were selected for this study: two species of *Comastoma* (*C. falcatum* and *C. traillianum*) are annual, while the other two species of *Lomatogonium* (*L. longifolium* and *L. perenne*) are perennial. In China, there are approximately 417 species of Gentianaceae belonging to 20 genera, which are commonly distributed in high-elevation ecosystems in southwestern China, including the Qinghai-Xizang plateau and its neighboring regions (Flora of China; http://www.iplant.cn/foc). As a prominent family in mountain ecosystems, Gentianaceae has long captivated botanists, ecologists, and plant enthusiasts who appreciate their vibrant flowers.

During a scientific expedition in late September 2023, seven populations were selected from the alpine ecosystems of the high Hengduan Mountains in southwestern China (**Supplementary Figure S1**). By chance, three populations of *C. traillianum*, two populations of *L. longifolium*, one population each of *C. falcatum* and *L. perenne* were specifically chosen (**Supplementary Table S1**). In each population, we endeavored to collect individuals of the target species from four distinct microhabitats, namely under shrub plants (referred to as shrub-engineered microhabitat), within cushion canopies (referred to as cushion-engineered microhabitat), within sedge grass patches (referred to as tussock grass-engineered microhabitat), and in bare ground screes (vegetation cover less than 5%) (**Figure 1**). In total, we collected data for all species in bare ground, shrub- and tussock grass-engineered microhabitats across most populations, while only collected data on cushion-engineered microhabitats for *C. traillianum* in the ZDS population (**Supplementary Table S1**).

Like many species in the Gentianaceae family, all target species for this study exhibit late flowering and were in bloom when collected in the field. However, most of the other plants present in our study populations were already at fruiting stage or even beginning to wither, including those engineering plants found in each type of microhabitat. Consequently, it was challenging for us to specifically identify all the engineering plants within shrub- and grass-engineered micro-habitats; we thus only classified them at a genus level. However, all engineering species observed within shrub habitats belonged to R*hododendron* species, while those within grass habitats were exclusively sedge plants (**Figure 1**).

### 2.2 Sampling and data collection

In each study population, we conducted random walks and collected 12 individuals of the target species from different microhabitat types, respectively. To excavate the individuals, a pick-mattock was used to carefully remove a *ca.* 10 cm × 10 cm × 15 cm soil block with the stem base of the target individual as the center of its diagonal lines. The surrounding soils around the root systems were then meticulously removed, and any soils adhering to the fine roots were gently brushed off. After complete removal of all soils, we recorded the number of flowers and leaves for each individual and measured their height (from caudex to apex). Subsequently, each individual was placed in a paper envelope and labeled with relevant information. All envelopes from one microhabitat type in each population were consolidated into a large plastic zip-lock bag containing sufficient silica gel desiccants to facilitate rapid drying. On returning to the laboratory, all samples were further dried at 70°C for 48 h.

After drying, each sampled individual was divided into the following four functional groups: reproductive organs (flowers), photosynthetic organs (leaves), supportive structures (stems) and water and nutrient absorptive systems (roots). The weights were measured at an accuracy of 0.1 mg. The combined mass of flowers, leaves, and stems was considered as the above-ground mass, while the root mass was designated as the below-ground mass. Furthermore, the total mass of leaves, stems, and roots was referred to as vegetative mass. Reproductive effort (RE) was calculated as the ratio between the total mass of the flowers and the total individual mass expressed as a percentage. The root fraction (RF) represented the proportion of the root mass in relation to the total individual mass, also expressed as a percentage. The average flower/leaf mass denotes the division between the total flower or leaf masses by their respective counts.

### 2.3 Data analysis

It was suggested that when using linear mixed-effect models, for practical purposes, there must be a reasonable number of random-effects levels – more than 5 or 6 at a minimum (Bolker, 2023), a criterion that our data did not satisfy. More importantly, our main objective in this study was to detect the influences of ecosystem engineers on resource allocation strategies in associated plants. Accordingly, we only adopted one-way ANOVAs to examine the impact of microhabitat on individual height, individual mass, flower/leaf number, average flower/leaf mass, stem mass, reproductive effort (RE), and root fraction (RF) for populations in which three distinct microhabitat types were sampled. The microhabitat was treated as an independent variable. For populations where individuals were only collected from two microhabitats, we employed independent-sample t tests to compare the relevant differences in the aforementioned parameters between microhabitats. Additionally, to assess whether resource allocation strategies differ among populations of the same species (*i.e.*, across different environmental backgrounds), data specifically related to *C. traillianum* and *L. longifolium* were extracted separately. And two-way ANOVAs were then performed with microhabitat and population as factors to test their effects on the above-mentioned parameters, which served as dependent variables. Shapiro-Wilk test and Q-Q plot were simultaneously employed to check the normality of the dataset. The data was subjected to a log10 transformation if they did not meet the assumptions of parametric statistics.

To detect the potential impact of individual size (total mass) on the resource allocation strategy, we employed linear regression analyses to determine the relationships between RE, RF, and individual size. Furthermore, in order to explore potential trade-offs among different functional organs, we conducted correlation analyses to examine the bivariate relationships involving: reproductive and vegetative masses; average flower mass and flower number; average leaf mass and leaf number; below-ground (root) mass and above-ground mass. We fitted the regressions on a log-log scale as proposed by the allometric biomass partitioning theory and model (Weiner, 2004; Weiner et al., 2009) that log *Y_1_* = log *β* + α log *Y_2_*, where *β* is the allometric constant, α the scaling exponent, and *Y_1_* and *Y_2_* the interdependent variables for allocations in certain plant parts.

All the above analyzes were performed using R 4.3.2 software (R Core Team, 2023).

## 3 Results

### 3.1 Impacts of ecosystem engineers on the growth of associated plants

**Figure 2:**
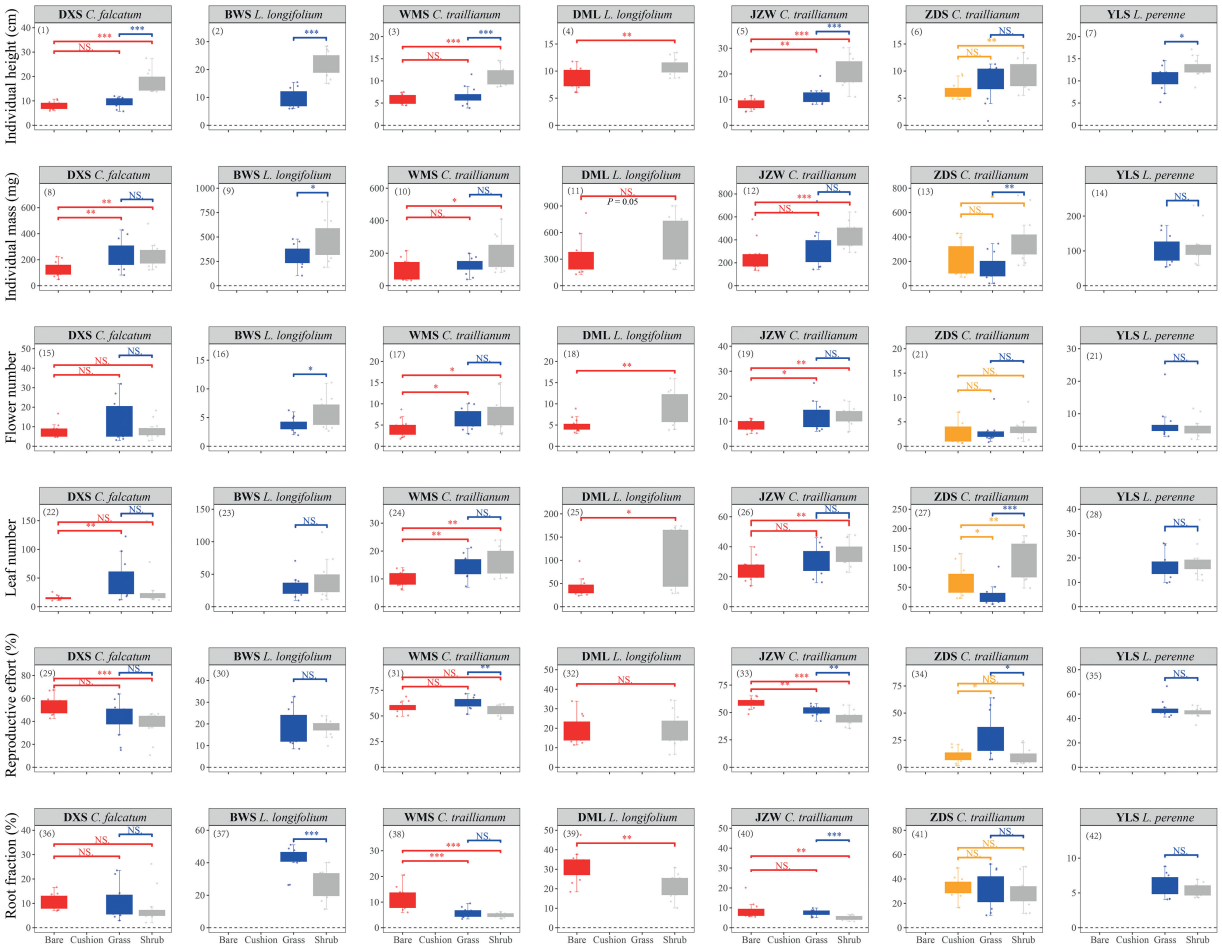
Individual height (1–7), total individual dry biomass (8–14), flower number (15–21), leaf number (22–28), reproductive effort (29–35) and root fraction (36–42) of target species established within specific microhabitats in the indicated populations. Statistical significance was indicated by ^*^, ^**^ and ^***^ for *P* < 0.05, *P* < 0.01, and *P* < 0.001, respectively; while NS indicated no significance. Similar notation was used in other figures.

The microhabitat type had significant effects (marginally significant for *L. longifolium* in the DML population; *P* = 0.054) on both individual height and mass for all study species in all populations, except for *L. perenne* mass parameters in the YLS population (**Table 1**; **Figure 2**). Specifically, individuals established within shrub-engineered microhabitats exhibited the highest individual height, followed by those in tussock grass-engineered microhabitats, while individuals in bare ground habitats showed the shortest height [**Figure 2** (1–7)]. Similarly, individuals within engineered microhabitats generally displayed higher biomass accumulations, either in terms of total individual mass [**Figure 2** (8–14)] or stem mass [**Supplementary Figure S2** (1–7)], compared to their counterparts in bare ground habitats (**Table 1**). Overall, shrub -engineered microhabitats exerted the strongest positive effects on plant height and biomass accumulation, followed by sedge grass- and cushion-engineered microhabitats, both of which showed similar effects (**Figure 2**, **Supplementary Figure S2**). However, the results of two-way ANOVAs conducted separately on *C. traillianum* and *L. Longifolium* indicated that apart from microhabitats, population also significantly influences resource allocation strategies (**Supplementary Table S2**).

**Table 1:** Results from one-way ANOVAs or independent-sample t -tests examining the impact of microhabitats on the resource allocation strategies of target species.

Tested parameter	Study population/Species																				
	DXS/*C. falcatum*			BWS/*L. longifolium*			WMS/*C. traillianum*			DML/*L. longifolium*			JZW/*C. traillianum*			ZDS/*C. traillianum*			YLS/*L. perenne*		
	One-way anova			Independent-sample t text			One-way anova			Independent-sample t text			One-way anova			One-way anova			Independent-sample t text		
	df	F	*P*	df	t	*P*	df	F	*P*	df	t	*P*	df	F	*P*	df	F	*P*	df	t	*P*
Individual mass	2	5.69	**0.008**	14.87	-2.70	**0.017**	2	4.20	**0.024**	18.76	-2.06	0.054	2	5.76	**0.007**	2	7.70	**0.002**	20.26	-0.05	0.963
Individual height	2	46.37	**< 0.001**	20.82	-7.83	**< 0.001**	2	27.30	**< 0.001**	21.08	-2.99	**0.007**	2	39.01	**< 0.001**	2	4.00	**0.028**	21.34	-2.56	**0.018**
Stem mass	2	9.62	**< 0.001**	21.87	-5.69	**< 0.001**	2	8.52	**0.001**	13.43	-2.93	**0.011**	2	10.99	**< 0.001**	2	5.18	**0.011**	21.99	-0.49	0.627
Flower number	2	3.07	0.060	16.07	-2.60	**0.019**	2	3.86	**0.031**	18.38	-3.30	**0.004**	2	4.76	**0.015**	2	0.49	0.619	17.95	0.74	0.468
Leaf number	2	6.69	**0.004**	17.43	-1.38	0.186	2	7.24	**0.002**	17.50	-2.57	**0.019**	2	2.34	0.112	2	19.86	**< 0.001**	16.51	0.44	0.667
Average flower mass	2	2.23	0.124	21.84	-1.32	0.202	2	1.23	0.306	17.60	0.66	0.515	2	3.52	**0.041**	2	0.79	0.461	19.81	-2.19	**0.041**
Average leaf mass	2	6.63	**0.004**	16.02	-1.08	0.297	2	1.63	0.212	21.69	-0.16	0.871	2	3.03	0.062	2	2.45	0.102	20.75	-0.38	0.707
Reproductive effort (RE)	2	4.06	**0.027**	17.02	-0.33	0.746	2	5.75	**0.007**	21.63	-0.20	0.842	2	21.61	**<0.001**	2	7.13	**0.003**	17.97	1.57	0.135
Root fraction (RF)	2	0.60	0.558	21.74	4.60	**< 0.001**	2	26.94	**< 0.001**	21.03	3.50	**0.002**	2	6.80	**0.003**	2	0.38	0.685	17.54	1.75	0.097

Study populations are abbreviated as follows and remain consistent across all figures and tables: DXS (Daxueshan snow mountains), BWS (Bowashan snow mountains), WMS (Wumingshan snow mountains), DML (Demula snow mountains), JZW (Jianziwan snow mountains), ZDS (Zheduoshan snow mountains), and YLS (Yelashan snow mountains). Statistical significance with P < 0.05 was highlighted by bold values.

Overall, there were no significant differences in average flower mass between individuals of different microhabitats; however, individuals of *C. falcatum* in the JZW population produced larger flowers in shrub-engineered and bare ground microhabitats compared to those of the sedge grass microhabitat; and individuals of *L. perenne* produced larger flowers in the shrub-engineered microhabitat compared to those in the sedge grass microhabitat [**Table 1**; **Supplementary Figure S2** (8–14)]. Furthermore, all species had a similar average leaf mass, except for individuals of *C. falcatum* in the DXS population, which produced larger leaves within the shrub-engineered microhabitat compared to those in the grass and bare ground microhabitats [**Table 1**; **Supplementary Figure S2** (15–21)]. Engineered microhabitats generally resulted in higher flower and/or leaf production compared to bare ground habitats. However, there was no significant difference in the number of flowers or leaves produced between grass- and shrub-engineered microhabitats [**Figure 2** (15–28)]. Only individuals of *L. longifolium* in the BWS population showed significantly higher flower production within the shrub-engineered microhabitat compared to the grass-engineered microhabitat; similarly, individuals of *C. falcatum* in the ZDS population exhibited significantly higher leaf production within the cushion- and shrub-engineered microhabitats than within the grass-engineered microhabitat [**Figure 2** (15–28)].

### 3.2 Impacts of ecosystem engineers on reproductive effort of associated plants

The reproductive efforts (RE) of the two annual plants were significantly higher than those of the two perennial plants, except for *C. traillianum*, which exhibited the lowest RE in the ZDS population (two-way ANOVA result for species effect: *df* = 3, *F* = 39.78, *P* < 0.001). Furthermore, the two annual species (*C. falcatum* and *C. traillianum*) generally exhibited higher RE in bare ground microhabitats compared to their counterparts within engineered micro-habitats; meanwhile, the two perennial species (*L. Longifolium* and *L. perenne*) demonstrated similar RE between microhabitats [**Table 1**; **Figure 2** (29–35)]. Similarly, individuals of all target species exhibited reduced root fractions within engineered microhabitats compared to their counterparts in bare ground microhabitat, although not all differences reached statistical significance [**Table 1**; **Figure 2** (36–42)].

**Figure 3:**
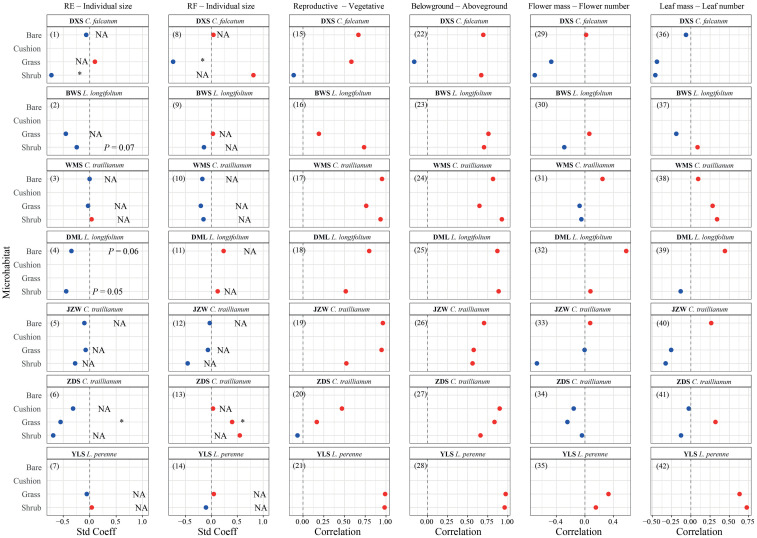
Standardized coefficient of regressions between reproductive effort, root fraction, and individual size (1–14), and Pearson’s correlation coefficient between each pair of functions for target plants in specific microhabitats in indicated populations (15-42).

Generally, a negative relationship between RE and individual size was observed. However, significant relationships (*P* < 0.05) were only observed for *C. falcatum* individuals within shrub-engineered microhabitats in the DXS population and for *C. traillianum* individuals within grass-engineered microhabitats in the ZDS population [**Figure 3** (1–7)]. Marginally significant results were observed for *L. longifoliumin* within the shrub-engineered microhabitat (*P* = 0.07) in the BWS population, as well as within both the shrub-engineered microhabitats (*P* = 0.05) and bare ground habitat (*P* = 0.06) in the DML population [**Figure 3** (1–7)]. Furthermore, although there was variation in the relationship between root fraction and individual size among species, microhabitats, and populations, it was generally non-significant (*P* > 0.05). Significantly negative relationships were only observed for *C. falcatum* in the DXS population within grass-engineered microhabitats; whereas positive relationships were found for *C. traillianum* in the ZDS population also within grass-engineered microhabitats (**Figure 3** (8–15)].

### 3.3 Impacts of ecosystem engineers on trade-off patterns of associated plants

The correlations between reproductive and vegetative allocations [**Figure 3** (15–21)] and between above-ground and below-ground allocations [**Figure 3** (22–28)] were consistently positive, implying no trade-offs between these functions. However, it is important to note that these correlations may be influenced by species-specific, population-specific, and microhabitat-specific attributes. Specifically, for *C. falcatum*, a significantly positive correlation between reproductive and vegetative allocations was only observed in the bare ground microhabitat; whereas significantly positive correlations between above-ground mass and below-ground mass were found in both the shrub-engineered microhabitat and the bare ground habitat (**Figure 3**). For *C. traillianum*, significant positive correlations between reproductive and vegetative mass were observed in all microhabitats of WMS and ZDS populations; however, these correlations were not significant for all microhabitats within the ZDS population (**Figure 3**). However, the correlation between below-ground and above-ground mass remained consistently positive across all microhabitats except for the shrub-engineered microhabitat within the JZW population (**Figure 3**). In the case of *L. longifolium*, the correlations between reproductive and vegetative mass, as well as those between belowground mass and aboveground mass, were significantly positive across all habitats except for grass-engineered microhabitat within BWS population [**Figure 3** (15–28)]. Finally, in the case of *L. perenne*, significant positive correlations existed between reproductive and vegetative traits, as well as between below-ground and above-ground biomass across all microhabitats [**Figure 3** (15–28)].

The correlations between average flower mass and flower number [**Figure 3** (29–35)], as well as between average leaf mass and leaf number [**Figure 3** (36–42)] exhibited a generally negative trend within engineered microhabitats but a positive trend in bare ground habitats. However, these correlations are also contingent upon species, population, and microhabitat types. For instance, individuals of *C. falcatum* within shrub- and grass-engineered microhabitats in the DXS population showed negative correlations between flower mass and number, whereas a positive correlation was observed in the bare ground habitat [**Figure 3** (29)]. Furthermore, *C. traillianum* individuals within engineered microhabitats displayed a negative correlation between leaf mass and leaf number; however, a positive correlation was found in the bare ground habitat in the JZW population [**Figure 3** (40)]. All these results collectively suggest that trade-offs may contextually exist in our target species.

## 4 Discussion

Our key findings demonstrate that ecosystem engineers possess the ability to modulate resource allocation strategies of other associated plants, albeit with potential influence from environmental backgrounds of populations. Importantly, these modulations are contingent upon both the type of engineered microhabitat and the life history strategies of plants (*i.e.*, annuals and perennials). These findings not only enhance our comprehension of plant adaptation to local environments but also provide insights into the persistence mechanisms of plant populations in alpine ecosystems.

### 4.1 Impacts of ecosystem engineers on the growth of associated plants

Engineered microhabitats can significantly enhance plant growth by either increasing individual height or promoting dry biomass accumulation (**Figure 2**, **Supplementary Figure S2**). This suggests that individuals established within engineered microhabitats exhibit faster growth rates compared to their conspecific counterparts in bare ground habitats. These findings support our first hypothesis and align with previous studies that demonstrate the facilitative role of ecosystem engineers (Badano and Cavieres, 2006a, 2006b; Cavieres et al., 2007; Anthelme et al., 2014; Ballantyne and Pickering, 2015; Chen et al., 2015b, 2020b; Ramírez et al., 2015). Such facilitation may be particularly crucial for annual plants like the *Comastoma* species studied here, as they need to complete their life cycles within a short growing season in alpine ecosystems where enhanced growth and resource accumulation confer advantages for successful reproductive outcomes. Additionally, the successful establishment, survival, and subsequent reproduction of seedlings may significantly influence the persistence and distribution of plant populations (Harper, 1977; Fortini et al., 2022). The underlying mechanisms behind this facilitation involve ecosystem engineers directly or indirectly modulating resource availability and ameliorating climatic factors for other species, thereby providing relatively safer microhabitats with abundant resources for other plants (Jones et al., 1994). Unfortunately, this study did not investigate how different ecosystem engineers modify microenvironments, thus preventing a direct correlation between microenvironmental attributes and target plant performance. However, previous studies have repeatedly confirmed the ecosystem engineering effects of ecosystem engineers in our study region (Yang et al., 2010; Chen et al., 2015a, 2015b, 2019, 2020b), including on the high Qinghai-Xizang plateau (Li et al., 2011; Pugnaire et al., 2015; Jiang et al., 2018; Zhao, 2023; Liu et al., 2023). Based on these facts, we believe that the involved engineered microhabitats indeed provide suitable living conditions for our target species.

We observed a general decrease in the root fraction (RF) within engineered microhabitats compared to bare ground habitats [**Figure 2** (36–42)], supporting our third hypothesis. According to the optimal partitioning hypothesis (Bloom et al., 1985; Chapin et al., 1987), plants allocate more resources to leaves in high-nutrient or moisture environments and shift biomass toward roots under low-nutrient or moisture conditions (McConnaughay and Coleman, 1999). As evidenced by previous studies, engineered microhabitats exhibit higher nutrient and/or moisture levels than bare ground habitats (Cavieres et al., 2007; Ramírez et al., 2015; Chen et al., 2015b), thus alleviating below-ground stressors. Consequently, plants established within these engineered microhabitats do not require additional resource investment to withstand below-ground stresses, allowing reallocation of saved resources towards above-ground functions. This reallocation may result in increased flower and/or leaf production, as indicated by our results [**Figure 2** (15–28)]. Although the average flower or leaf mass did not differ significantly among microhabitats [**Supplementary Figure S2** (8–21)], both engineered microhabitats exhibited higher numbers of flowers or leaves compared to bare ground habitats [**Figure 2** (15–28)]. Furthermore, stem mass was also greater within engineered microhabitats [**Figure 2** (1–7)]. The presence of more flowers/leaves or stronger stems can have important implications for plant reproduction and persistence. On the one hand, more flowers imply higher reproductive potentials (*e.g.*, seed production) assuming a consistent seed set among flowers within the same population. On the other hand, a greater number of leaves may imply enhanced photosynthetic productivity given similar photosynthetic efficiency across leaves. Additionally, frequent strong winds in alpine environments can also exert pressures on plant survival (Körner, 2003; Nagy and Grabherr, 2009); therefore, possessing sturdy stems could enhance their resistance to such winds and subsequently increase individual survival rate. Collectively, these findings suggest that individuals established within engineered microhabitats possess advantages compared to their counterparts in bare ground habitats, either in terms of reproductions (by means of flowers) or individual persistence.

### 4.2 Impacts of ecosystem engineers on reproductive effort of associated plants

Surprisingly, the reproductive effort (RE) of the two annual plants within engineered microhabitats was significantly lower compared to that in bare ground habitats; however, the RE of the two perennial plants exhibited similar patterns between microhabitats [**Figure 2** (29–35)]. These findings contradict our second hypothesis and indicate that the saved resources from earlier root investment were not reallocated prior to reproduction. Furthermore, irrespective of the effect of microhabitats, the REs of the two annual plants were significantly higher than those of the two perennial plants, except for *C. traillianum* which displayed the lowest RE in the ZDS population (two-way ANOVA result for species effect: *df* = 3, *F* = 39.78, *P* < 0.001). This result aligns with previous studies confirming that annual plants allocate a higher proportion of energy/biomass to reproduction compared to perennial plants (Brock, 1983; Iwasa and Cohen, 1989; Vico et al., 2016). Possible explanations include that annual plants reproduce only once within a single growing season and therefore may allocate more resources to reproduction to maximize fitness since there is no need to conserve resources for future use (Harper, 1977; Reekie and Bazzaz, 2005). On the other hand, due to their prolonged life cycle allowing multiple reproductions over several seasons, perennial plants may prioritize resource allocation toward stress-resistant functions before allocating them toward reproduction later on or in subsequent year (s) (Reekie and Bazzaz, 2005). Perennial plants allocate resources to storage structures such as roots, rhizomes, and stems for extended survival under adverse conditions (Bloom et al., 1985; Bazzaz et al., 1987). The trade-off between life history stages in perennial plants (Reekie and Bazzaz, 2005) might result in a decreased average reproductive investment per year, thereby providing an explanation for the higher RE observed in annual species compared to perennial species in this study. Consequently, the reduced RE of annual plants within engineered microhabitats, as opposed to their counterparts in bare ground habitats, suggests that while they benefit from certain aspects facilitated by ecosystem engineers, they may concurrently experience additional stresses that require resource allocation for resistance purposes as discussed subsequently.

One question that arises is why the resources saved from root investment are not proportionally allocated to reproductive function, particularly for annual plants. It is widely acknowledged that plant growth in natural communities is limited by multiple resources, leading to intricate resource allocation strategies (McCarthy and Enquist, 2007; Umaña et al., 2021). For example, Umaña et al. (2021) observed an increase in leaf allocation under soil nitrogen limitation at the expense of nonphotosynthetic tissues and a greater allocation to stems when both above-ground and below-ground resources (light and soil phosphorus) were co-limited at the cost of roots. In our study on plants established within engineered microhabitats, particularly shrub-engineered microhabitats, it should be noted that while ecosystem engineering effects may alleviate soil nutrient- or moisture-related stresses, light stress can still be significant due to shading by shrub canopies (Lei et al., 2006). Light availability plays a crucial role in plant resource allocation strategies (Xie et al., 2014; Umaña et al., 2021). Individuals experiencing light stress under shrub or tussock canopies may allocate more resources towards taller stems and/or increased leaf production in order to enhance light competition, resulting in reduced reproductive effort. Therefore, we propose that light stress significantly influences the resource allocation patterns of our focal species within engineered microhabitats. Collectively, it seems that the impact of ecosystem engineers on plant resource allocation strategies is contingent upon both below-ground (nutrient and moisture) and above-ground (light) resources. The interactive effects of these two components likely give rise to contrasting responses in terms of above-ground versus below-ground biomass allocations, as well as reproductive versus vegetative biomass allocations.

Size-dependent reproductive allocations are commonly observed in plants, with RE either increasing or decreasing with individual size (Hemborg and Karlsson, 1998; Pino et al., 2002; Méndez and Karlsson, 2004). However, these size-dependent effects are often influenced by various environmental factors that exhibit temporal and spatial variability (Schmid and Weiner, 1993; Hemborg and Karlsson, 1998; Guo et al., 2012). For example, Hemborg and Karlsson (1998) found a decrease in the RE of *Trollius europaeus* (Ranunculaceae) with increasing individual size at lower elevations but no size-dependency was observed at higher elevations. Our results indicate a general negative relationship between REs of our target species and individual size; however, only a few engineered microhabitats showed (marginally) significant relationships as described in the results section [also see **Figure 3** (1–7)]. These findings suggest that as plant individuals become larger, their REs may decrease while ecosystem engineers might amplify this negative effect. Given the available data in this study, elucidating the precise underlying mechanisms and processes remains challenging. Nonetheless, it is worth noting that while engineered microhabitats may provide favorable living conditions, such as enriched nutrients and moisture, for promoting individual biomass accumulation (*i.e.*, larger size), the shading effect caused by ecosystem engineers’ canopies might necessitate additional resources to enhance stem strength and/or leaf production, consequently leading to reduced reproductive efforts. Consequently, negative relationships between reproductive effort and individual size could potentially be observed within engineered microhabitats. Further studies should be conducted to explore how such patterns are influenced by surrounding environments in particular populations.

### 4.3 Impacts of ecosystem engineers on trade-off patterns of associated plants

Empirical studies have consistently demonstrate trade-offs in plant resource allocation processes (Forbis and Doak, 2004; Vilela et al., 2008; Du and Qi, 2010; Buckley and Avila-Sakar, 2013; Rosa-Schleich et al., 2019). For example, increased investment in root systems can lead to decreased investment in above-ground modular organs (Umaña et al., 2021). Our findings indicate a generally positive correlation between vegetative and reproductive allocations, as well as between underground (root system) and above- ground allocations [**Figure 3** (15–28)]. Furthermore, the correlations between average flower/leaf mass and flower/leaf number were predominantly neutral with only a few exceptions observed within engineered microhabitats (**Supplementary Figure S2**). These results indicate that trade-offs among these different functions may be context- and species-specific-dependent, further supporting our argument that interactive effects of below-ground resources such as nutrients and moisture, along with above-ground resources such as light intensity, contribute to complex resource allocation patterns exhibited by our target plants within engineered microhabitats.

## 5 Summary and perspective

In conclusion, this study provides evidence that ecosystem engineers can regulate the resource allocation strategies of other associated plants, possibly by creating favorable microhabitats characterized by reduced environmental severity and increased resource availability. Generally, plants established within engineered microhabitats allocate more resources to above-ground functions while reducing allocation to below-ground function (*i.e.*, roots). Although reproductive efforts are lower within engineered microhabitats, individuals exhibit larger size and higher flower, leaf, and stem production. These modulations may enhance individual survival and reproductive potential, thus favoring population persistence in severe alpine environments. Consistent with previous studies (Gomez-Aparicio, 2009; Anthelme et al., 2014), the findings of this study have potential implications for conservation, management and restoration practices in alpine ecosystems. For instance, ecosystem engineers can alleviate harsh micro-environments and thereby enhance the growth rate and survival of other associated plants. Consequently, they hold promise for accelerating vegetation recovery by facilitating new seedling establishment and population recruitment in damaged or degraded alpine ecosystems. However, since this study did not determine the specific mechanisms through which ecosystem engineers regulate resource allocation strategies of associated plants’, further research is required to elucidate how different stresses from below- and above- ground sources influence resource allocation strategies and comprehensively evaluate the contributions of ecosystem engineers to population dynamics of alpine plants.

## Data availability statement

The original contributions presented in the study are included in the article/**Supplementary Material**. Further inquiries can be directed to the corresponding authors.

## Author contributions

PY: Data curation, Formal analysis, Investigation, Writing – original draft. MN: Data curation, Formal analysis, Investigation, Writing – original draft. QF: Formal analysis, Visualization, Writing – review & editing. LQ: Formal analysis, Visualization, Writing – review & editing. MH: Investigation, Writing – review & editing. ZL: Investigation, Writing – review & editing. HS: Data curation, Formal analysis, Investigation, Writing – original draft, Writing – review & editing. JC: Conceptualization, Methodology, Supervision, Writing – original draft, Writing – review & editing, Formal analysis, Funding acquisition, Project administration.
