# Supplementary material for: Ecosystem engineers can regulate resource allocation strategies in associated plant species
Source: Front Plant Sci. 2024 Jun 6;15:1387951. doi: 10.3389/fpls.2024.1387951 (PMC11187262; doi:10.3389/fpls.2024.1387951)
Supplement: Supplementary file 1 [file DataSheet_1.docx]

**Supplementary materials**

**Supplementary Table 1.** Information of selected species, populations and relevant micro-habitat types sampled in each population. DXS: Daxueshan snow mountains; BWS: Bowashan snow mountains; WMS: Wumingshan snow mountains; DML: Demula snow mountains; JZW: Jianziwan snow mountains; ZDS: Zheduoshan snow mountains; YLS: Yelashan snow mountains. Abbreviations for study populations are consistent through the whole text, tables and figures.

| Study population |  | Geographical information |  | Elevation (m) |  | Species |  | Life history |  | Sampling micro-habitat |
| --- | --- | --- | --- | --- | --- | --- | --- | --- | --- | --- |
| DXS |  | N28°34′17″  E99°48′26″ |  | 4346 |  | *Comastoma falcatum* |  | Annual |  | Shrub + Grass + Bare |
| BWS |  | N28°53′5″  E100°18′45″ |  | 4595 |  | *Lomatogonium longifolium* |  | Perennial |  | Shrub + Grass |
| WMS |  | N29°8′22″  E100°3′39″ |  | 4678 |  | *Comastoma traillianum* |  | Annual |  | Shrub + Grass + Bare |
| DML |  | N29°19′19″  E97°1′57″ |  | 4774 |  | *Lomatogonium longifolium* |  | Perennial |  | Shrub + Bare |
| JZW |  | N30°1′41″  E100°52′13″ |  | 4333 |  | *Comastoma traillianum* |  | Annual |  | Shrub + Grass + Bare |
| ZDS |  | N30°5′05″  E101°28′24″ |  | 4499 |  | *Comastoma traillianum* |  | Annual |  | Shrub + Grass + Cushion |
| YLS |  | N30°10′33″  E97°19′47″ |  | 4616 |  | *Lomatogonium perenne* |  | Perennial |  | Shrub + Grass |

**Supplementary Table 2.** Results of two-way ANOVA for testing the effect of study population and micro-habitat on the resource allocation strategies of target species. Note: the effect of interaction was ignored.

| Tested parameter | *C. traillianum* | | | | | |  | *L. longifolium* | | | | | |
| --- | --- | --- | --- | --- | --- | --- | --- | --- | --- | --- | --- | --- | --- |
|  | Tested factor | | | | | | | | | | | | |
|  | Study population | | | Micro-habitat | | |  | Study population | | | Micro-habitat | | |
|  | df | F | *P* | df | F | *P* |  | df | F | *P* | df | F | *P* |
| Individual height | 2 | 26.4 | < 0.001 | 3 | 22.75 | < 0.001 |  | 1 | 50.17 | **< 0.001** | 2 | 33.06 | **< 0.001** |
| Individual mass | 2 | 25.81 | < 0.001 | 3 | 10.41 | < 0.001 |  | 1 | 0.004 | 0.95 | 2 | 5.69 | **0.006** |
| Average flower mass | 2 | 14.59 | < 0.001 | 3 | 12.3 | < 0.001 |  | 1 | 6.84 | **0.01** | 2 | 4.85 | **0.01** |
| Reproductive effort (RE) | 2 | 88.75 | < 0.001 | 3 | 63.34 | < 0.001 |  | 1 | 0.03 | 0.87 | 2 | 0.13 | 0.88 |
| Root fraction | 2 | 169.33 | < 0.001 | 3 | 45.82 | < 0.001 |  | 1 | 2.16 | 0.15 | 2 | 16.61 | **< 0.001** |
| Stem mass | 2 | 35.01 | < 0.001 | 3 | 13.57 | < 0.001 |  | 1 | 11.78 | **0.001** | 2 | 22.26 | **< 0.001** |
| Flower number | 2 | 56.26 | < 0.001 | 3 | 15.99 | < 0.001 |  | 1 | 3.20 | 0.08 | 2 | 4.65 | **0.01** |
| Leaf number | 2 | 62.03 | < 0.001 | 3 | 35.05 | < 0.001 |  | 1 | 8.69 | **0.005** | 2 | 5.69 | **0.006** |
| Average leaf mass | 2 | 1.58 | 0.21 | 3 | 4.36 | 0.006 |  | 1 | 0.05 | 0.83 | 2 | 0.81 | 0.45 |
| Root : shoot ratio (R-S-R) | 2 | 144.90 | < 0.001 | 3 | 40.27 | < 0.001 |  | 1 | 2.16 | 0.15 | 2 | 18.84 | **< 0.001** |


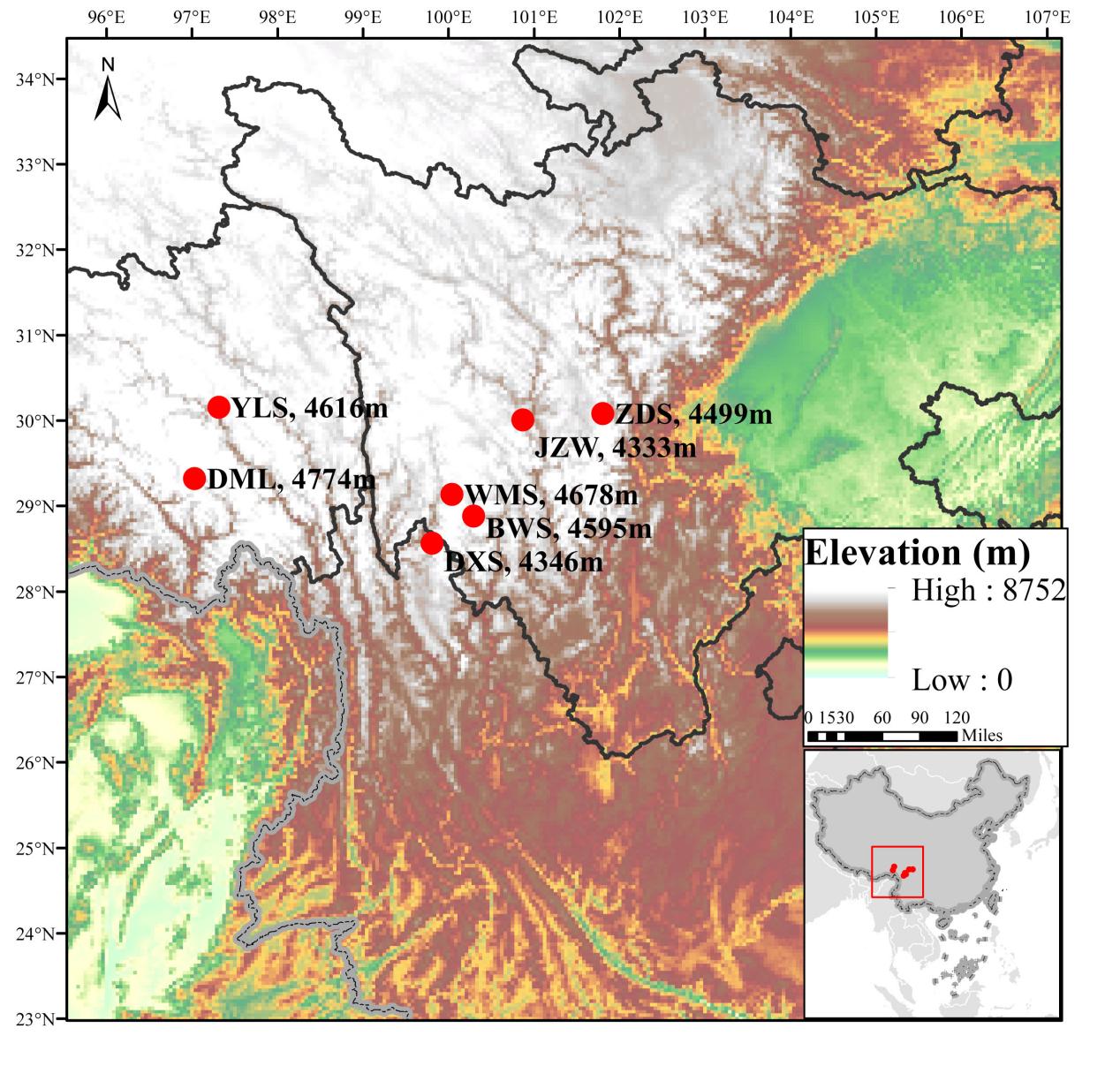


**Supplementary Figure 1.** Study area and populations. The abbreviations for the study populations are as follows: DXS - Daxueshan snow mountains, BWS - Bowashan snow mountains, WMS - Wumingshan snow mountains, DML - Demula snow mountains, JZW - Jianziwan snow mountains, ZDS - Zheduoshan snow mountains, and YLS - Yelashan snow mountains. Refer to Table S1 for detailed information on the study population and species.


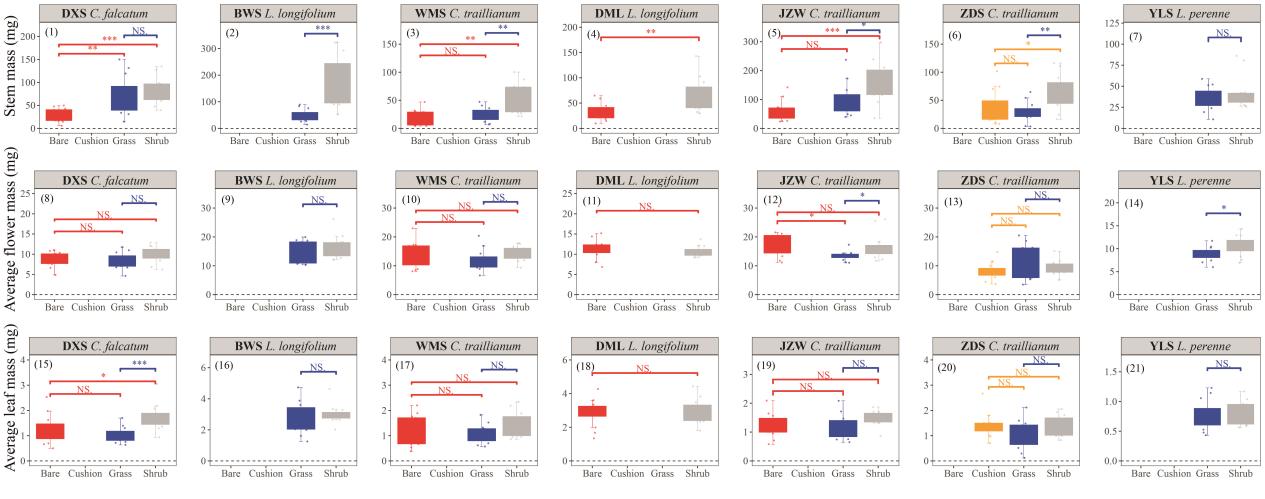


**Supplementary Figure 2.** Stem, average flower and leaf mass of target species established within specific micro-habitats in indicated populations.
